# RETRACTION: Targeting MYCN IRES in MYCN‐amplified Neuroblastoma With Mir‐375 Inhibits Tumor Growth and Sensitizes Tumor Cells to Radiation

**DOI:** 10.1002/1878-0261.13775

**Published:** 2024-12-03

## Abstract

**RETRACTION:** H. Zhang, T. Liu, S. Yi, L. Gu, and M. Zhou, “Targeting MYCN IRES in MYCN‐amplified Neuroblastoma With Mir‐375 Inhibits Tumor Growth and Sensitizes Tumor Cells to Radiation,” *Molecular Oncology* 9, no. 7 (2015): 1301–1311, https://doi.org/10.1016/j.molonc.2015.03.005.

The above article, published online on 24 March 2015 in Wiley Online Library (wileyonlinelibrary.com), has been retracted by agreement between the journal Editor‐in‐Chief, Kevin Ryan; FEBS Press; and John Wiley & Sons Ltd. The retraction has been agreed upon following an investigation into concerns raised by a third party, which revealed inappropriate image section duplications within the article (Figure 5B) and between this (Figures 3B and 4B) and another article published by the same group of authors in a different scientific context. The authors were unable to provide a satisfactory explanation and the original raw data, which has led the editors to lose confidence in the data presented. Therefore, the editors consider the conclusions substantially compromised and are retracting the paper.

**RETRACTION:** H. Zhang, T. Liu, S. Yi, L. Gu, and M. Zhou, “Targeting MYCN IRES in MYCN‐amplified Neuroblastoma With Mir‐375 Inhibits Tumor Growth and Sensitizes Tumor Cells to Radiation,” *Molecular Oncology* 9, no. 7 (2015): 1301–1311, https://doi.org/10.1016/j.molonc.2015.03.005.

The above article, published online on 24 March 2015 in Wiley Online Library (wileyonlinelibrary.com), has been retracted by agreement between the journal Editor‐in‐Chief, Kevin Ryan; FEBS Press; and John Wiley & Sons Ltd. The retraction has been agreed upon following an investigation into concerns raised by a third party, which revealed inappropriate image section duplications within the article (Figure 5B) and between this (Figures 3B and 4B) and another article published by the same group of authors in a different scientific context. The authors were unable to provide a satisfactory explanation and the original raw data, which has led the editors to lose confidence in the data presented. Therefore, the editors consider the conclusions substantially compromised and are retracting the paper.

